# Familiarity affects other-regarding preferences in pet dogs

**DOI:** 10.1038/srep18102

**Published:** 2015-12-16

**Authors:** Mylene Quervel-Chaumette, Rachel Dale, Sarah Marshall-Pescini, Friederike Range

**Affiliations:** 1 Comparative Cognition, Messerli Research Institute, University of Veterinary Medicine, University of Vienna, 1 Veterinärplatz, 1210 Vienna, Austria; 2 Wolf Science Center, Dörfles 48 – 2115 Ernstbrunn, Austria

## Abstract

Other-regarding preferences are considered to be the foundation of human cooperation. However, the evolutionary origin of this behavior in humans remains poorly understood. So far, comparative studies in primates have led to mixed conclusions probably due to methodological differences relating to both task complexity and the types of control conditions used. Moreover, no clear link between phylogenetic relatedness and prosociality has been found, suggesting that other convergent selection pressures may play a role in the evolution of such behaviors. Here, using one of the cognitively less demanding tasks, we show for the first time, that dogs can behave pro-socially by donating food to a conspecific partner, but only if the partner is familiar. This highlights the importance of considering the social relationships between individuals when testing animals for other-regarding behaviors. Moreover, by including a social control condition, we show that the dogs’ prosocial response was not due to a simple social facilitation effect. The current findings support recent proposals that other convergent selection pressures, such as dependence on cooperative activities, rather than genetic relatedness to humans, may shape a species’ propensity for other-regarding behaviors.

Compared to the rest of the animal kingdom, human large-scale cooperation is exceptional and considered to be at the origin of our unusual cognition, technology and culture^1^,^2^,^3^,^4^,^5^,^6^,^7^. One of the prerequisites required for such cooperation is thought to be other-regarding preferences between individuals^6^,^7^. Pro-social behaviors, or other-regarding preferences, are defined as voluntary actions that benefit others^8^. So far researchers have mainly investigated the presence of other-regarding preferences in our closest relatives (but see^9^,^10^), with some studies, but not all, finding positive results (see^11^ for review).

Interestingly, the comparative studies in primates show no clear link between phylogenetic closeness to humans and pro-social tendencies^11^,^12^, suggesting that other convergent selective pressures may drive other-regarding preferences. Indeed, some have suggested that reliance on cooperative activities (e.g. collaborative foraging^13^, cooperative breeding^6^,^14^) or strong social bonds^15^ might have been the driving force behind the selection for other-regarding preferences across species. Since cooperative activities are not confined to primates, a wider comparative approach is necessary to better understand whether cooperative traits might have played a role in the evolution of pro-social behaviors.

So far, studies investigating pro-sociality in non-primates has been limited to rats and jackdaws^9^,^10^. Canids are also potentially good models for a comparative approach because of their high level of sociability and the presence of cooperative behaviors in pup-rearing, joint territorial defense and group hunting in a number of species^16^,^17^,^18^,^19^,^20^. Furthermore, domestic dogs have been selected for special social skills during domestication^21^,^22^, which could have further enhanced their propensity to show pro-social behaviors. Indeed a study with pet dogs showed that subjects helped a human to retrieve a desirable object by pressing a button. However, this behavior was only evident when the human gave strong communicative cues by pointing at the button itself^23^, suggesting that dogs may have perceived the context as an ‘obedience’ task. Removing human participation from the experimental context may help in better assessing dogs’ other-regarding preferences. Therefore in the current study, we adopted a simplified version of a classic prosocial choice paradigm ‘the bar-pulling task’, in which a dog could choose whether to deliver food to their conspecific partner in an adjacent enclosure.

As well as expanding the range of species studied, our study also aimed to counter some limitations evident in previous studies using the bar-pulling paradigm. Indeed, in most studies that used this particular prosocial choice paradigm, only one control condition, where the partner is not present at all in the adjacent enclosure, was compared to the test condition. Therefore, any observed pro-social responses during the test could have been triggered by the mere presence of a partner rather than other-regarding preferences (e.g. desire for social contact^24^, and/or local enhancement effects^10^). Importantly, in the current study we included a social facilitation control condition, as has been used in other paradigms with non-human primates^25^,^26^,^27^,^28^, in which the partner was present but unable to access the reward.

Furthermore, some authors have argued that task complexity could explain why some studies do not find pro-social responses in primates^29^,^30^. Consequently, rather than using the more commonly adopted 1/1 vs. 1/0 food distribution, we based our paradigm on a cognitively more simple food distribution already used in several primate studies^14^,^25^,^31^,^32^, where the donor could choose between 0/0 and 0/1 food distribution, therefore only having to keep track of the location of one piece of food.

Finally, one common difficulty for all studies investigating pro-social behaviors is finding the balance between allowing animals enough experience to understand the mechanism of the task, yet avoiding over-training that could bias the results during the test. Therefore, differently from previous studies, here we took advantage of the training process and used it as a pillar of our experiment. By training all animals extensively and then looking at the extinction of the previously reinforced behavior (i.e. pulling the giving tray) within different conditions, we made sure that the condition rather than the training *per se* drove the response of the animals.

Specifically, in our set-up donor dogs (n = 16), had the choice of pulling a tray delivering food to the enclosure next to it (receiver enclosure) but none to itself (0/1) or a tray which delivered food to neither self nor the other enclosure (0/0) (Fig. 1). If the donor pulled the giving tray, it provided food to the conspecific receiver. Furthermore, since we did not know whether the pulling action alone could be self-rewarding, the empty tray (0/0) offered the dogs the strategy to stop delivering the food without ceasing to pull.

Dogs carried out a single session of each of five experimental conditions (presented in a semi-random counterbalanced order, see methods section for more details); in the familiar test condition (F.test), a familiar partner dog from the same household was placed in the receiver enclosure. In the stranger test condition (S.test), a stranger partner (of the same sex as the familiar partner), with whom the donor had no prior interaction, sat in the receiver enclosure (Fig. 2a). Additionally, we conducted three control conditions: i) a familiar social facilitation control (F.SFC), in which the familiar partner sat one meter away on the other side of the donor enclosure, no longer having access to the food, ii) a stranger social facilitation control (S.SFC) where the stranger dog was present but could not access the food (Fig. 2b) and iii) a non-social control (NSC), in which the partner enclosure was empty and no other dog was in the testing room (Fig. 2c). Importantly none of our donors served as receivers.

Before each trial, the experimenter asked the donors to sit on a start location from where they could witness the baiting process. In order to avoid any communicative cues, the experimenter baited the tray through holes in a curtain placed in front of the bar-pulling apparatus, whilst remaining enclosure, and dogs had five seconds to pull one of the two ropes (overall mean latency of pulling ± s.e: 1.15 seconds ± 0.05). The baited tray was semi-randomized such that it was never the same tray more than twice in a row.

A session ended either if the donor did not pull on five consecutive trials or if they refused to move to the start location prior to the next trial, despite five consecutive requests. Importantly, shortly after the session ended, the experimenter conducted four “knowledge probe” trials where the food was randomly placed either on the top or on the bottom tray in front of the donor enclosure. If donors understood the location of food delivery, in these trials they should start pulling the bar again because the food is delivered to their own enclosure.

In order to look at the extinction rate of giving pulls, control and test sessions were interspersed (on separate days) with motivation sessions where the door between the two enclosures was open and the donor had to successfully retrieve the food from the receiver enclosure after pulling the giving tray on 17 out of 20 trials. Importantly, this criterion ensured that donor dogs regained their initial level of motivation prior to each control/test session. Therefore, even though subjects participated in five sessions where they were unrewarded, by including motivation sessions between each, the number of trials, pulls and giving pulls performed by the donors were compared across control/test sessions.

## Results

Since the number of trials, total pulls (both 0/0 and 0/1) and giving pulls (0/1) were highly correlated (see supplementary information) and dogs hardly ever pulled the empty tray (0/0, mean range across conditions ± s.e.m., n = 16 individuals: 0.6 to 1.5 ± 0.34), in the following we report only the number of trials where donors pulled the giving tray since this best reflects the actual food delivered by the donor.

Results showed that the pulling of the giving tray decreased from the first to the last session independently of condition (likelihood ratio test: χ^2^ = −13.129; p < 0.001, Fig. S1). Indeed, despite the fact that donors regained their initial level of motivation before entering in each control/test session, as can be expected in an extinction paradigm, an overall decrease in their pulling rate was observed. This is likely due to the fact that in the initial sessions the animals’ expectation of getting a reward was still higher due to their prior, solely positive experience of receiving rewards when pulling.

However, despite this session effect, large differences in the giving responses were still found between conditions (Fig. 3). When paired with a familiar receiver, donors gave more food than when the receiver was a stranger (F. test: mean ± s.e = 20.5 ± 1.9; S.test: mean ± s.e = 8.9 ± 1.9 ; glmm: z = −4.26; p < 0.001 after Bonferroni correction; Fig. 3). Importantly, dogs also pulled the giving tray more for the familiar partner in the test than in both the non-social control (NSC: mean ± s.e = 13.3 ± 2.4 ; glmm: z = 2.09, p < 0.05) and the social facilitation control, when the familiar partner was close to the donor but had no access to the reward (F.SFC: mean ± s.e = 12.9 ± 2.5 ; glmm: z = −2.39, p < 0.05; Fig. 3). Additionally, there were no differences between the non-social control condition (NSC) and the social facilitation controls (glmm_F.SFC_: z = −0.24, p = 0.81; glmm_S.SFC_: z = 0.04, p = 0.97; S.SFC: mean ± s.e = 13.2 ± 1.6). These three control conditions represent the baseline rate of giving pulls performed by the donors when they were not being rewarded for pulling (hence in some respects it measures to what extent pulling the rope is self-rewarding in itself). The fact that donors pulled the giving tray significantly more with the familiar receiver than in these control conditions confirms that dogs were capable of distinguishing between conditions in which the partner received food, and when they did not. Hence, their giving behavior was not driven by a simple social facilitation response.

Interestingly, donors actually pulled more in both the non-social and the stranger social facilitation control, when no one was being rewarded, than in the test when the stranger dog could access the food (glmm_NSC_: z = −2.30, p < 0.05 ; glmm_S.SFC_: z = −2.25, p < 0.05, see Fig. 3).

Importantly, no interaction between the session order and condition emerged, showing that the dogs delivered more food to the familiar partner than the stranger and pulled the giving tray more often with the familiar partner than in all control conditions independently of when the test session was conducted.

Behavior coding further demonstrated that the unwillingness to give food to the stranger was not due to discomfort in their presence, as there was no influence of the condition on the amount of stress or agonistic behaviors exhibited by donors (see supplementary information for details on behavior coding, statistical analyses and results). Moreover, at the end of each session in “knowledge probe” trials when dogs could pull the tray for themselves, all dogs pulled on 100% of trials (see supplementary information, Fig. S3). These results further confirm that dogs understood the contingencies of the task and that the lack of pulling behavior when the stranger was present in the receiver enclosure was not due to a decreased willingness to approach the apparatus.

Another possibility is that donors pulled less with the stranger because they were occupied interacting with him/her. However, we rarely observed donors sniffing or interacting with the stranger dog during the test (see supplementary information). Moreover, these interactions mostly occurred when the partner first entered the room and not during the session itself. In the rare cases it happened during the test, the experimenter waited for the end of these interactions before starting the next trial. Therefore, the overall lack of pulling when dogs were paired with the stranger was not a by-product of donors being too occupied with interacting with the stranger partner to focus on the task.

## Discussion

By using a simplified version of the classic prosocial bar-pulling paradigm, we found a significant difference in the extinction rate of giving across conditions, with donors working for longer and consequently providing more food for a familiar receiver than a stranger.

Moreover, the similar extinction rate of giving across the three control conditions confirms that dogs understood that the food was inaccessible to both themselves and the partner in all three situations. It hence provides a good baseline measure of dogs’ unrewarded pulling inclination, allowing us to compare it to conditions in which a reward was delivered to the partner. Interestingly, donors extended their usual extinction rate of giving when paired with a familiar receiver and, on the contrary, inhibited their tendency to pull the giving tray when a stranger dog received the food.

Despite our social facilitation controls, some could argue that donors are simply seeking contact with their familiar partners, consequently being closer to the apparatus and thus, incidentally more likely to pull the rope. Three lines of evidence suggest this was not the case: 1) before starting a new trial, the experimenter asked the donor to sit on the start location, located furthest from the partner. If donors were seeking contact with the familiar partner, one would have expected that they were unwilling to move away from the partner to sit on the start location and consequently should have stopped the session earlier in the familiar test than in the other conditions when the familiar partner was not present in the receiver enclosure, but this was not the case; 2) since in the social facilitation control the partner was closer to the start location, donor dogs should have remained on the start location and hence pulled the apparatus less in this condition than in the non-social control when no partner was present in the room, but again this was not the case; 3) the three control conditions did not differ from each other in terms of the donor’s rate of pulling the giving tray, suggesting that the donor’s proximity to the partner (familiar, stranger and no partner at all) was irrelevant in influencing the pulling rate of donors.

Another argument could be that dogs were ‘worried’ about the presence of the stranger and hence were less likely to approach the apparatus placed adjacent to them when they were in the receiver enclosure, or they were ‘curious’ about the stranger and hence too busy investigating it to focus on the task. Again these two options can be excluded since 1) stress behaviors were rare and did not differ across conditions, 2) aggressive behaviors towards the stranger partner were extremely rare (N = 4), and therefore statistical analyses could not be performed (see supplementary information for more details); 3) during ‘knowledge probes’ at the end of each session, when the food was placed so that the donor could reach it after pulling, the rate of ‘pulling for oneself’ was at 100% whether a stranger or familiar partner was in the receiver enclosure, showing that dogs were neither too ‘wary’ nor too ‘curious’ about the stranger to manipulate the apparatus if they themselves received the food.

Alternatively, we know that humans and non-human primates are more inequity averse towards familiar individuals with whom they have a lower relationship quality (Humans^33^, Chimpanzees^34^). In dogs, this was not found to be the case^35^. However, relationship quality in the dog study was measured via owner questionnaire rather than direct observation of the animal’s behavior, and in dog and primate studies, inequity responses with strangers have not been tested. Therefore, a stronger inequity aversion towards a stranger rather than a familiar individual cannot be rejected as an explanation for the dogs’ unwillingness to donate to a stranger in our experiment. Indeed, this inequitable situation might explain why the donation rate is higher in the control conditions than in the stranger test.

We can hence conclude, not only that the response we see is pro-social, but also that dogs show a greater tendency to exhibit those behaviors towards familiar social partners than strangers. These findings support the hypothesis that other-regarding preferences in non-humans is affected by the social relationship between individuals^11^,^36^.

One potential ultimate explanation for the difference in dogs’ donation rate according to the partner’s identity could be due to both dog’s ancestry and their natural ecological life style. Indeed, wolf society is characterized by communal pup raising, territorial defense and cooperative hunting^18^,^19^,^20^. Although dogs in a free-ranging setting no longer form pair-bonds and allo-maternal care seems to be reduced^16^, they continue to live in social packs, and coordinate defense of their territory towards out-group members^18^. Hence dogs’ ancestry and continued dependence on other pack members for territorial defense may explain our result for other-regarding preferences biased towards conspecifics living in the same household. The current study cannot distinguish between in-group/out-group and familiarity bias, hence future work should investigate this possibility by assessing whether this bias against giving food to a stranger also holds for familiar out-group members.

At present little can be said about the proximate mechanisms driving pro-social bias towards familiar conspecifics. It may be due to the expectation of reciprocity from a familiar individual^12^. Alternatively, a simple form of empathy, such as emotional contagion^37^, may drive the response, whereby the positive emotion experienced by partners when they receive a reward may have a positive effect on the donor. Emotional contagion effects have been shown to be stronger if the partner is an in-group rather than out-group member^38^. A history of successful co-feeding may also influence the likelihood of prosocial responses in such a task, in that pet dogs habitually fed together may have an increased motivation to provide their partners with food, driven by an expectation of food becoming available also to themselves. It is important to note however that this latter mechanism may drive prosocial responses in such tasks in any social species routinely fed simultaneously in captivity, and/or also known to forage together in their natural environment.

Our finding that dogs can show prosocial behaviors towards conspecifics raises the question whether this is due to domestication^21^,^39^ or their wolf ancestry (canine cooperation hypothesis^40^) and highlights the need for such studies to be extended to a wider range of species. In fact, differently from what has been proposed, our results suggest that pro-social behaviors may not necessarily be linked to cognitive complexity^41^ and could have been shaped by a species’ reliance on cooperative activities (collaborative foraging hypothesis^13^ ; cooperative breeding hypothesis^6^) or a change in the emotional and socio-cognitive abilities brought about by domestication^21^,^39^. The simplicity in the cognitive demands of the current experimental paradigm may provide the ideal tool to test these hypotheses within a wider comparative framework.

## Methods

All procedures were discussed and approved by the institutional ethics committee in accordance with Good Scientific Practice guidelines and national legislation (Ref. 06/05/97/2013). The study took place at the Clever Dog Lab, Messerli Research Institute, University of Veterinary Medicine of Vienna in Austria.

### Training

Training comprised of two parts. In the first part, dogs were trained to sit on the ‘start location’ within the donor enclosure and received a reward for doing so. Separately, they were trained to pull on the rope to move the tray towards them, which resulted in a reward being delivered to the donor enclosure. Once these two behaviors were acquired separately, dogs moved on to the second step. In the second step, subjects were asked to sit on the start location and stay there whilst the experimenter baited the tray in front of the receiver enclosure. From this stage on, dogs were no longer rewarded for sitting on the start location. Once baiting was complete, the experimenter pushed the two trays forward simultaneously making the ropes available to the dog. During this training the door between donor and receiver enclosures was open, so if dogs selected the baited tray, they could go into the receiver enclosure and take the food from the tray. If they did not choose the baited tray, they were given 10 seconds to explore the enclosure before being asked to go back to the start location. During this step, the experimenter was hidden behind a curtain with only their hands visible during the baiting process. Dogs visited the lab twice a week during training. In order to proceed to the test, dogs had to successfully pull the baited tray and retrieve the reward in the receiver enclosure in 17 trials out of 20.

### Motivation sessions

Because our measure was the extinction of a previously rewarded behavior, in order to be able to directly compare our test and control conditions (see below), motivation sessions were run between each condition. In these sessions of 20 trials the donor dog was alone and the sliding door between the two enclosures was open, allowing the dog to retrieve the food in the receiver enclosure after pulling the baited tray. Dogs had to pull the baited tray on 17 out of 20 trials (probability of success = 0.85, p < 0.001) before they were allowed to proceed to the next test or control session, in order to demonstrate they had regained their initial level of motivation. All donors except one needed only one motivation session between each test/control condition. By using this method, we ensured the subjects returned to a high level of motivation to perform the task before entering the next test or control condition.

### Test and control conditions

Each donor participated in one session each of the two test and the three control conditions in a semi randomized and counterbalanced order in a way that the two conditions involving a particular partner (test condition and social facilitation control of familiar or stranger dog) were run one after each other. Moreover, we took care that half of our subjects experienced the familiar conditions before the stranger conditions and on the contrary, the other half started with the stranger conditions before the familiar. From our 16 donors, five received the non social control condition first, seven started with the test condition from which three were paired with the stranger partner and four with the familiar, and finally five donors received the social facilitation control conditions first from which three where paired with the familiar and two with the stranger. The non-social control was randomly positioned either at the start, between the stranger and familiar conditions or at the end.

**Figure 2:**
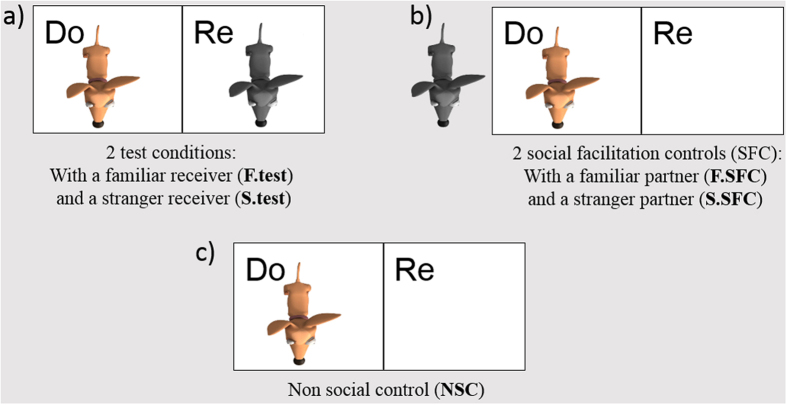
Location of the donor (Do) and receiver (Re) dog in each condition (figure drawn by MQC).

**Figure 3:**
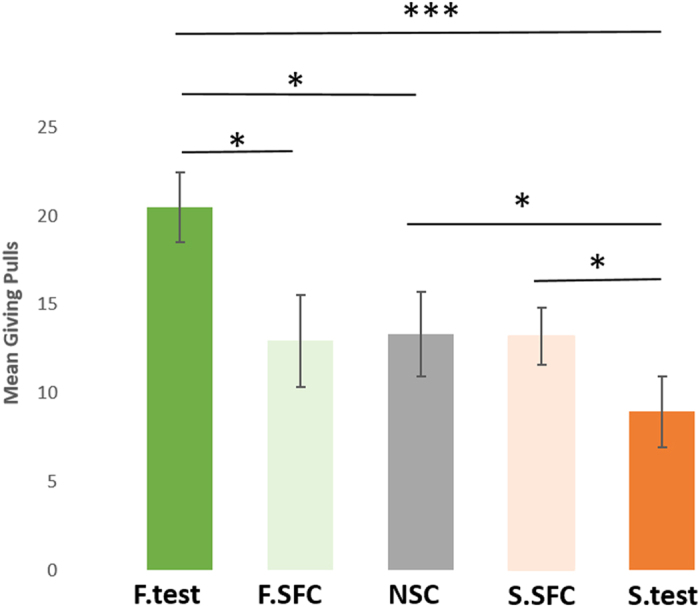
Mean number of giving pulls (mean ± s.e.m., n = 16 individuals) in which donors pulled the giving (0/1) tray across conditions (*p < 0.05, **p < 0.01, ***p < 0.001).

Both test and control sessions comprised a maximum of 40 trials each and only one session per day was carried out. A session ended either if the donor did not pull on five consecutive trials or if they refused to move to the start location prior to the next trial, despite five consecutive requests from the experimenter. At the end of each session (test and control) donor and partner (when present) remained in their original locations (Fig. 2) and we conducted four “knowledge probe” trials where pulling the baited tray now delivered food to the donor’s enclosure. These four trials ensured that dogs understood the contingency of the task and that even when paired with a stranger, dogs were still comfortable enough to manipulate the apparatus if they could obtain food for themselves. All subjects always pulled on those four “knowledge probe” trials.

## Additional Information

**How to cite this article**: Quervel-Chaumette, M. *et al.* Familiarity affects other-regarding preferences in pet dogs. *Sci. Rep.* **5**, 18102; doi: 10.1038/srep18102 (2015).

## Supplementary Material

Supplementary Information

Supplementary video

## Figures and Tables

**Figure 1:**
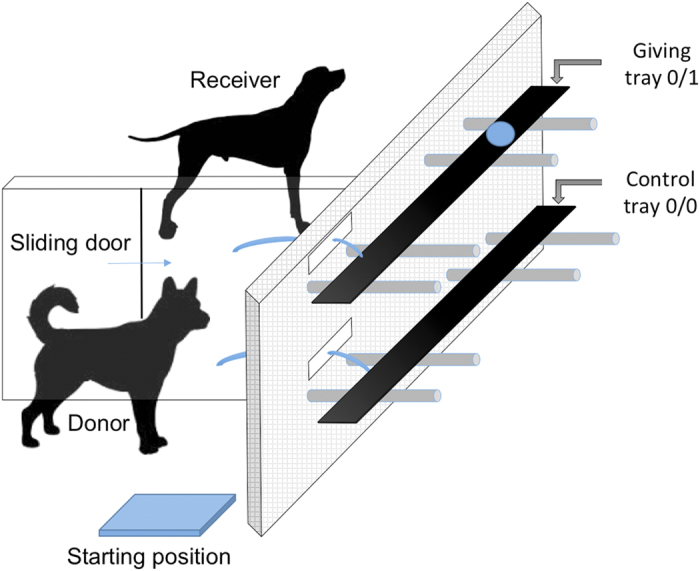
Side view of the bar-pulling apparatus (figure drawn by MQC).
