# Supplementary material for: Familiarity affects other-regarding preferences in pet dogs
Source: Sci Rep. 2015 Dec 16;5:18102. doi: 10.1038/srep18102 (PMC4680958; doi:10.1038/srep18102)
Supplement: Supplementary Information [file srep18102-s1.pdf]

Title: Familiarity affects other-regarding preferences in pet dogs

Authors: Mylene Quervel-Chaumette[1*], Rachel Dale[1,2*], Sarah Marshall-Pescini[1,2], Friederike Range[1,2]

Affiliations:

[1] Comparative Cognition, Messerli Research Institute, University of Veterinary Medicine, University of Vienna, 1 Veterinärplatz, 1210 Vienna, Austria.

[2] Wolf Science Center, Dörfles 48A – 2115 Ernstbrunn, Austria.

*Correspondence to:
Mylene Quervel-Chaumette
Comparative Cognition, Messerli Research Institute, University of Veterinary Medicine, University of Vienna, Vienna, Austria.
1 Veterinärplatz,
1210 Wien
Austria
Tel: 0043-69917366265
mylene.chaumette@vetmeduni.ac.at

Rachel Dale
Tel : 0043-69918332188
rachel.dale@vetmeduni.ac.at

Supplementary Information

SI Results
All statistics were run in R (version 3.1.2 for windows)

Number of trials, pulls and giving-pulls.
Donors could end the session by either refusing to pull on five consecutive trials or refusing to sit on the start location despite five consecutive requests from the experimenter. Therefore, the first measure to consider was the total number of trials across conditions.
Then, within a trial, donors could decide to pull a tray or not to pull it. Furthermore, if they decided to pull, they could either pull the giving tray or the empty tray. Hence we also took into account the total number of pulls regardless of tray choice (i.e. including both giving pulls and empty pulls) and the number of giving pulls across conditions (i.e. pulling the tray that delivered food to the partner enclosure).
Preliminary analyses were carried out to investigate the potential correlation between these three dependant variables (Pearson's correlation test).
Results showed a strong correlation between the three possible dependant variables (see table S2), in that if donor dogs chose to participate in a trial they normally pulled a tray, and if they pulled a tray it was in most cases the baited tray (see Table S2 and Fig S1). Therefore the number of giving pulls was chosen as the dependent measure for further analyses as this quantifies the food delivered to the receiver enclosure and thus represents the best measure of other-regarding preferences.

Number of Giving-pulls
We used both AIC calculation and the likelihood ratio testing to assess the statistical influences of the various explanatory factors. In order to examine if the number of giving pulls differed across sessions and conditions, generalized linear models GLMM [31], correcting for over-dispersion by using the glmmADMB package and function, were used with the donor's identity as a random factor and the number of 'giving-pulls' as the response factor. The order of sessions (i.e. first to fifth, to check for order effects) and condition (familiar test, stranger test, familiar social facilitation control, stranger social facilitation control and non-social control) as well as the interaction between session and condition were included in the model as explanatory factors. The model revealed no interaction effect on the response variable. However, the order of sessions as well as the condition influenced the number of giving-pulls performed by the donor (see table S3, Fig S2 and Fig 3). We corrected for multiple testing by using a Bonferroni correction.

Percentage of success in the knowledge-probe trials
In order to ensure that dogs understood the contingency of the task and that even when paired with a stranger, dogs were still comfortable enough to manipulate the apparatus if they could obtain food for themselves, four knowledge-probe trials were run at the end of each test and control condition. In these four trials, the food was placed either on the top or bottom shelf in

front of the donor enclosure. This time, pulling the baited tray delivered food to the donor enclosure only. All dogs always pulled on knowledge-probe trials (Fig S3).

Other behaviors exhibited during testing

The video recordings of the test and control sessions were coded with Solomon Coder Beta 15.01.13 (Copyright András Péter, http://solomoncoder.com). We coded all occurrences of scratching, yawning, lips-licking and attempting to leave the donor enclosure and combined them in a single category dubbed 'stress behaviors'. The presence of agonistic behavior (growling, snapping and threatening) was coded as a binary variable (0 for none, 1 if it occurred in a session). Finally, we coded the frequency with which the partners (familiar and stranger) tried to reach the reward by, for example scratching at the fence/apparatus (See table S4 for details of the ethogram adopted). The behavior coding was done by one author (MC) and twenty percent of the videos (N = 16) were coded by a second observer (RD). Cronbach's alpha coefficient was used to measure the level of agreement. For all variables and behaviors the alpha was between 0.86 and 1, which corresponds to a high level of agreement [32].

Stress behaviors

Linear mixed models using the lme function and the packages "lme4" were run with the identity of the donor as a random effect, the percentage of duration of "stress behaviors" as response factors and session and conditions as fixed factors. The statistical significance of the full model was tested by comparing it to a null model ("conditions" excluded) by using a likelihood ratio test (R function "anova" see table S3).

The comparison between the two models did not result in a significant reduction of fit. This indicates that the condition did not influence the frequency of stress behaviors exhibited by the donors (likelihood ratio test: χ^2=3.68; p=0.45).

Agonistic behaviors

Four dogs growled once when paired with the stranger dog in the test condition but no agonistic behaviors occurred in the other conditions rendering statistical comparison across conditions impossible. Overall, donor dogs did not show agonistic behaviors towards partners. The receiver dogs did not show any agonistic behaviors.

Reaching for the food

Finally, a Pearson's correlation test between the number of giving pulls and the frequency of reaching for the food by the partner in the test was used to examine whether donors were more likely to provide food when the partner tried to reach the food.

In both test conditions (familiar and stranger), no significant correlations were found between reaching for the food and the number of giving pulls (F.test: r_s=0.25 $mean_{reach\ for\ food}$ = 9.3, $mean_{Giving}$ = 20.4; S.test: r_s=0.17, $mean_{reach\ for\ food}$ = 9.3, $mean_{Giving}$ = 8.9 ; n=16).

SI Tables (S1-S4)

Table.S1. Age, sex and breed of the donors and receivers participating in the experiment

Donor's name	Donor's Sex	Donor's Age	Donor's breed	Number of training session before testing	Familiar partner's name	Familiar Partner's Age	Familiar Partner Breed	Stranger partner's Name	Stranger partner's Breed	Receive[illegible] Partne[illegible] Sex
Finn	Male	11	Australian Shepherd	4	Juno	3	Australian Shepherd	Lola	Mixed breed	Female
Luke	Male	10	Border Collie	2	Quismo	8	Border Collie	Buck	Beagle	Male
Joker	Male	8	Border Collie	2	Ally	7	Sheltie	Lola	Mixed breed	Female
Ultimo	Male	6	Border Collie	2	Sokrates	9	Bardino-Podenco Canario-Mix	Buck	Beagle	Male
Talie	Male	4	Husky	4	Luna	2	Husky	Lola	Mixed breed	Female
Casper	Male	3	Border Collie	2	Ninni	2	Irish Wolfhound	Lola	Mixed breed	Female
Mago	Male	11	Golden Retriever	2	Tika	8	Husky-Golden Retriever mix	Lola	Mixed breed	Female
Bounty	Female	9	Australian Shepherd	2	Juno	3	Australian Shepherd	Lola	Mixed breed	Female
Achuck	Female	8	Chesapeake bay retriever	2	Elrond	5	Chesapeake bay retriever	Michel	Mixed breed	Male
Chio	Female	8	Border collie	2	Sonic	7	Terrier	Michel	Mixed breed	Male
Neela	Female	7	Australian Shepherd	3	Leni	2	Australian Shepherd	Lola	Mixed breed	Female
Chasie	Female	6	Border Collie	2	Gatsby	3	Border Collie	Buck	Beagle	Male
Faye	Female	6	Border Collie	2	Ini	11	Border Collie	Lola	Mixed breed	Female
Mali	Female	6	Mixed breed	3	Jacob	11	Parson Russell Terrier	Michel	Mixed breed	Male
Sunny	Female	3	Mixed breed	2	Lizzy	12	West Highland White Terrier	Lola	Mixed breed	Female
Flappi	Female	6	Pumi-Mixed breed	2	Joey	6	Mixed breed	Buck	Beagle	Male

Table.S2. Matrix of Pearson's correlation coefficient between the three potential dependant variables.

**Pearson's correlation coefficient**	**Number of trials**	**Number of total pulls**	**Number of giving pulls**
Number of trials	NA	NA	NA
Number of total pulls	R=0.95	NA	NA
Number of giving pulls	R=0.95	R=0.99	NA

Table. S3. AIC value for model selection and likelihood ratio tests comparing final reduced and full models

Variable		AIC	
Number of Giving Pulls	Full model	540.6	
	Model1: Interaction out	536.2	
	Session out	546.3	
	Condition out	547.3	
Number of Giving Pulls	Model comparison	Chi Square	p-value
	Model 1 versus Full model	4.1408	=0.459
	Session out versus Model 1	13.129	<0.001
	Condition out versus Model1	47.960	<0.001
Stress Behavior	Condition out versus Model 1	5.2145	=0.256

Table.S4. Ethogram.

Behaviors		Definition
Stress behaviors	Yawning	Opening the mouth wide and then closing it.
	Lips-licking	To lick the lips
	Attempt to leave enclosure	The body is orientated toward the exit. The nose is touching the end of the fence. Dogs may be pushing the fence with the head or pawing at it or simply standing in front of the exit.
	Scratching	Scratching any part of the body
Agonistic behaviors	Threat	Subject orients towards another performing one or more of the following: staring at, curling of the lips, baring of the canines, raising the hackles, snarling, growling, and barking, sometimes with the tail perpendicular or above the back.
	Snapping	To snap teeth into the air, noisily.
Reaching for the food	….	The partner scratches the front fence of the enclosure or puts the paw through the fence towards the tray

SI Figures (S1-S3)

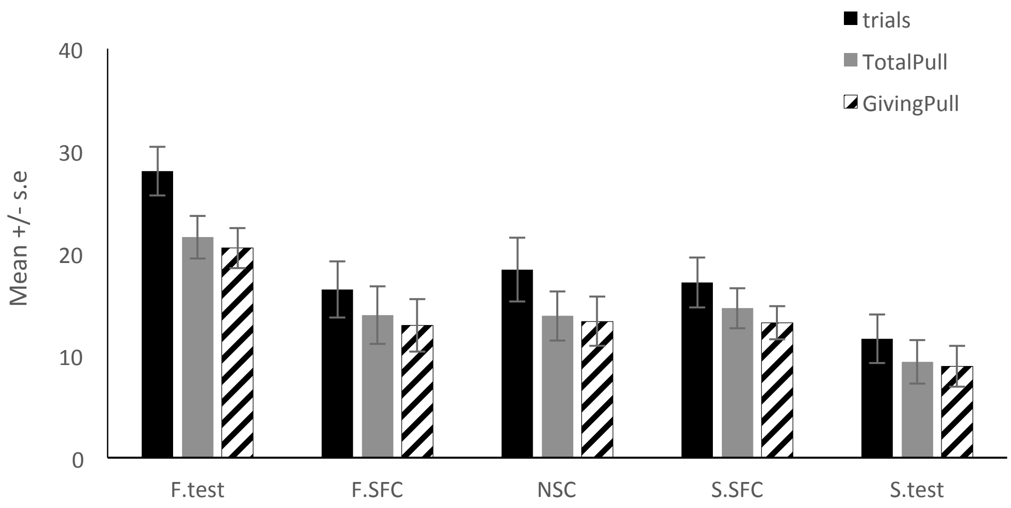

Fig.S1. Mean number of trials, pulls and giving pulls ± s.e (standard error bars) performed by the donors across conditions.

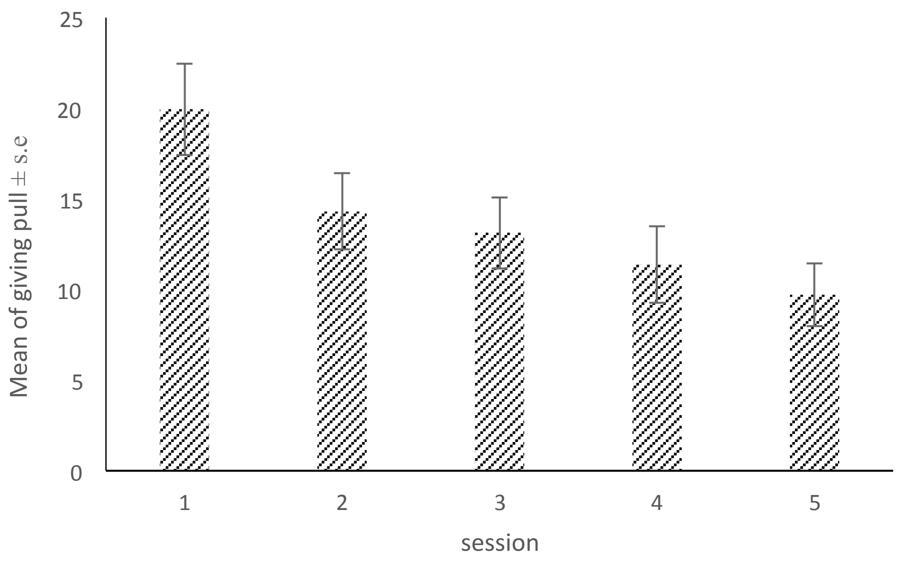

Fig.S2. The number of giving pulls decreases across session (session (likelihood ratio test: χ^2=-13.129; p<0.001).

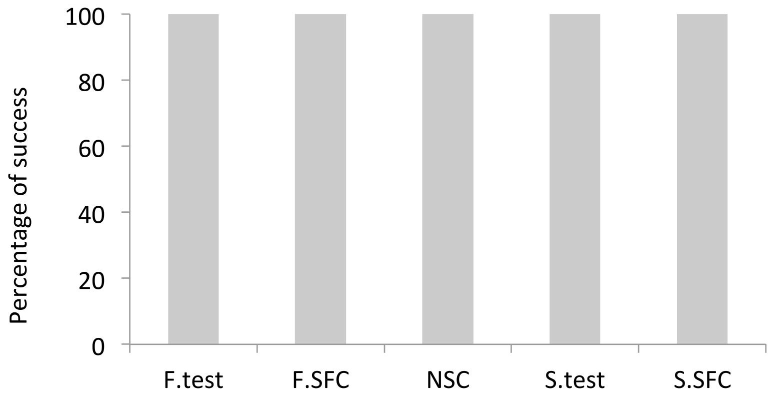

Fig.S3. Percentage of pulling the baited tray during the knowledge-probe trials by the donors at the end of each condition.

SI Movie

Video clip demonstrating each of the conditions: a) motivation control. b) stranger test, c) stranger social facilitation control, d) familiar test, e) familiar social facilitation control and f) non-social control.

Supplemental References (31-32)

31. Fournier DA, Skaug HJ, Ancheta J, Lanelli J, Magnusson A, Maunder M.N, Nielsen A, Sibert J (2012). AD Model Builder: using automatic differentiation for statistical inference of highly parameterized complex nonlinear models. *Optim. Methods Softw.* 27:233–249.

32. Nunnally IH, Bernstein JC (1994) Psychometric theory (3nd ed.). *PsycCRITIQUES.* 24:275-280.
